# Co‐Designing Culturally Tailored Bowel, Cervical and Breast Cancer Screening Interventions With Migrant Communities: Towards Closing the Migration‐Related Cancer‐Equity Gap in New South Wales, Australia

**DOI:** 10.1111/hex.70805

**Published:** 2026-08-05

**Authors:** Andre M. N. Renzaho, Sheetal Challam, Nina Hartcher, Mitchell Smith, Sandra Elhelw, Amanda Jayakody, Sarah McGill, Tracey A. O'Brien

**Affiliations:** ^1^ Translational Health Research Institute, School of Medicine Western Sydney University Campbelltown Campus Campbelltown New South Wales Australia; ^2^ Bridging Divides Scholar of Excellence Toronto Metropolitan University Toronto Ontario Canada; ^3^ Cancer Institute NSW St Leonards New South Wales Australia; ^4^ South Western Sydney LHD, NSW Refugee Health Service Liverpool New South Wales Australia; ^5^ Multicultural NSW Harris Park New South Wales Australia

**Keywords:** bowel cancer screening, breast screening, cervical screening, co‐design, migrants, nominal group technique, refugees

## Abstract

**Background:**

Migrants in Australia experience persistent cancer‐equity gaps, with later‐stage diagnoses linked to low screening participation. This study aimed to co‐design cancer screening interventions with migrant and refugee communities to promote equitable participation and reduce disparities in bowel, cervical and breast cancer screening in New South Wales, Australia.

**Methods:**

Using stratified convenience sampling, 90 first‐generation Middle Eastern and Sub‐Saharan African migrants across five NSW regions participated in 10 face‐to‐face nominal group technique sessions to elicit and rank intervention priorities.

**Results:**

Participants identified 24 priority actions for bowel cancer screening, 11 for breast, and eight for cervical cancer, grouped into intervention domains. Bowel cancer drew the broadest response, spanning cancer literacy, policy reform, community action, and monitoring and evaluation. Breast cancer priorities centred on literacy and policy, while cervical cancer input focused on literacy and behavioural confidence. Increasing cancer literacy, shifting behaviours and enacting supportive policy reform emerged as consistent priorities across all three cancers.

**Conclusion:**

Improving migrant and refugee participation across all three screening programs requires culturally grounded interventions that build cancer literacy and leverage trusted networks including peers, community champions and frontline health workers. Settlement programs, community organisations and schools provide critical infrastructure for sustained screening education, while policy reforms including age threshold reductions, incentive mechanisms and improved access support are essential to achieving equitable outcomes.

**Patient or Public Contribution:**

Migrants with lived experience, cancer caregivers and community champions played a central role in setting priorities and co‐designing intervention options.

## 1 Introduction

Globally, 23.6 million new cancer cases and 10 million cancer deaths were reported in 2019 (representing a 26% increase in new cases, a 21% rise in deaths, and a 16% increase in disability‐adjusted life years since 2010 [1]. For the year 2019, cancer was the second leading cause of death worldwide, second only to cardiovascular diseases for the number of deaths, years of life lost and DALYs. These statistics mirror a trend observed in Australia. Cancer is responsible for around 3 of every 10 deaths in Australia [2]. There were 162,163 Australians diagnosed with cancer in 2022, which is a significant increase from around 87,922 cases of cancer in 2000, representing an increase of 84.4% in just over 22 years (or +3.84% per year). Similarly, from 2000 to 2022, the number of cancer deaths annually increased from 36,316 to 49,996 (+ 37.7%), whilst the age‐standardised cancer death rate decreased from 194.1 per 100,000 persons to 145.3 deaths per 100,000 persons per year (−25.1%) [2].

The decreasing cancer mortality rates translate into increased cancer survival rates due at least in part to the robust Australian Population‐Based Screening Framework [3, 4, 5]. Currently, Australia has three population‐based cancer screening programs aimed at reducing cervical, bowel and breast cancer mortality through early detection and treatment, and reducing cervical and bowel cancer incidence through the identification and treatment of precursors [3]. The National Cervical Screening Program, National Bowel Cancer Screening Program and BreastScreen Australia are of great public health significance because of their effectiveness in producing population survival benefits, significantly reducing their burden on the Australian health system [3]. In 2022, these three cancers accounted for 23.7% of all new cancer cases (9.7% for bowel cancer, 12.7% for breast cancer and 1.3% for cervical cancer) and 18.5% of all cancer deaths (11% for bowel cancer, 6.5% for breast cancer and 1% for cervical cancer) [4]. People with early detection for these three cancers through national cancer screening programs have comparatively high survival outcomes, with a 5‐year survival rate averaging 92% for breast cancer, 74% for cervical cancer and 71% for bowel cancer [4, 5].

However, the benefits of Australia's Population‐Based Screening Framework have been unequally distributed, with migrants and refugees experiencing significant disparities across the cancer care continuum. Key barriers include language limitations, low cancer literacy, unfamiliarity with the healthcare system, cultural distrust, fear of results and lack of female health professionals [6, 7, 8, 9]. Compared to Australian‐born populations, migrants from East Asia, Southeast Asia and North Africa/Middle East are 40%–60% less likely to screen for bowel cancer, those from North Africa/Middle East and East Asia are 50% less likely to screen for breast cancer, and migrant women from the Middle East and Asia are 12%–26% less likely to undertake cervical screening [10, 11, 12]. Migrants of African and Middle Eastern backgrounds consistently screen below recommended rates across all three cancers [11, 12].

These disparities are compounded by later‐stage diagnoses, and thus poorer outcomes, arising from non‐participation in early detection programs; yet most existing interventions have adopted a blanket approach to cancer prevention that fails to account for the distinct needs of migrant and refugee communities [13, 14, 15]. Evidence confirms that culturally tailored, community‐based programs improve screening behaviours and uptake, but such approaches remain underdeveloped and/or poorly implemented [16, 17, 18, 19]. Addressing these gaps necessitates co‐designed, culturally tailored, community‐based interventions. This study therefore aimed to co‐design such interventions with migrant and refugee communities to promote equitable participation and reduce disparities in bowel, cervical and breast cancer screening in New South Wales, Australia.

## 2 Materials and Methods

### 2.1 Design and Study Setting

This study was part of a larger research program examining cancer literacy and screening among the target population. The data presented in this study focus specifically on the identification of cancer‐related intervention priorities, generated through a co‐design process with community stakeholders [20]. The Nominal Group Technique (NGT) used pre‐determined questions to guide structured silent brainstorming, reducing peer influence and promoting equal participation. Recorded ideas were then discussed as a group to clarify and consolidate overlapping points, with duplicates removed. Anonymised voting to identify priorities limited the influence of both researchers and participants. This sequential approach, moving from problem identification to solution generation and priority ranking, enabled the production of a readily implementable, co‐designed action plan. Participants were asked to identify barriers to cancer screening uptake within their communities and propose feasible solutions to increase participation. The study protocol was approved by a steering committee comprising investigators, project management team members and partner organisation representatives, which met quarterly to provide high‐level guidance throughout the project [21, 22, 23, 24].

The NGT uses small‐group discussions with stakeholders with varying expertise, qualifications and influence to enable decision‐making and facilitate group consensus on complex yet sensitive topics like cancer prevention [21, 22, 23]. The approach incorporates idea generation and problem‐solving in the same session, creating an environment that allows contentious issues to be addressed collectively. Because the ranking gives equal weight to participants' voices and allows the effective management of dominant voices [25], it is valuable and robust when engaging refugee communities.

### 2.2 Sampling, Participant Recruitment and Procedures

Study participants were recruited using stratified convenience sampling. This method was the most appropriate due to the low literacy level of participants and the complex nature and sensitivity of discussing cancer. Convenience sampling enabled access to willing and accessible community members. To mitigate selection bias, samples were drawn from well‐defined subgroups stratified by country of birth, language and geographical location, capturing diversity across age, migration experience, migration status and lived experience.

Given the cultural diversity of our target communities, it was important to ensure cultural sensitivity throughout the engagement process. Cultural diversity is defined as ‘employing one's knowledge, consideration, understanding, respect and tailoring after realising awareness of self and others and encountering a diverse group or individual’ (p. 210) [26]. Our realisation of cultural sensitivity was driven by three principles: (1) the recognition of the importance of culture in the lives of our target communities, (2) acknowledging and respecting cultural differences and (3) minimising any negative consequences that might arise from cultural differences [26, 27, 28]. To address linguistic barriers, translated participant information sheets were developed and community leaders consulted to identify cultural barriers, maximise reach and inform community selection criteria. Once target communities and selection criteria were finalised, bilingual workers were recruited within those communities and trained in research ethics, eligibility criteria, confidentiality and informed consent. Recruitment was conducted through community groups and ethnic organisations via direct presentations and distribution of translated materials. Bilingual workers then contacted interested participants by phone or in person to arrange participation.

Eligibility criteria mirrored Australia's national cancer screening guidelines: participants were aged 50–74 years for bowel and breast cancer screening, and women aged 25–74 years for cervical cancer screening. Sub‐Saharan African and Middle Eastern migrant and refugee communities were the focus given their disproportionate burden across all three cancers, consistently lower screening uptake, higher rates of late‐stage diagnosis, and poorer survival outcomes compared to host populations [29, 30]. The study was conducted in New South Wales due to committed implementing partners, the State's focus on multicultural cancer screening, and available funding. Partner involvement ensured venues met occupational health and safety standards, accommodated participants' cultural and religious needs, and provided a spacious, well‐equipped environment conducive to independent thinking. This approach is particularly suited to collectivist contexts characterised by power imbalances, deference to elders, hierarchical communication, language barriers and trust issues, all of which can affect group dynamics and data quality [31, 32, 33, 34]. Given that collectivist values are prominent among Sub‐Saharan African and Middle Eastern migrants and refugees [31, 32], the NGT's structured processes effectively balance group‐oriented norms with equitable and independent contribution from all participants.

The research was co‐implemented with partner organisations working locally with target communities including Illawarra Multicultural Services, Metro Assist Inc., Mosaic Multicultural Connections, and NSW Service for the Treatment and Rehabilitation of Torture and Trauma Survivors (STARTTS). Through their community‐owned programs and bilingual workers, partner organisations carried out community mobilisation activities to map out and clearly define the target communities with low cancer screening participation rates, covering diverse participants across various cultural, religious and linguistic groups. Bi‐lingual workers of partner organisations were involved in designing the NGT schedule and standardising the NGTs' implementation guide to make the process consistent and culturally appropriate across all community groups. A discussion guide was developed with bi‐lingual workers of partner organisations to help facilitate discussions. The discussion guide outlined the purpose of the consultation, discussion objectives, and key health messaging across the three national cancer screening programs. Each session was pre‐faced with an education component to educate participants on bowel, breast and cervical cancer screening programs. The project received ethical approval from the South‐Western Sydney Local Health District Human Research Ethics Committee (2022/ETH01516).

### 2.3 Data Collection

Online training workshops on implementing an NGT were held for the bi‐lingual workers of the four agencies involved in local project delivery (27 April 2022). At the end of the training sessions, facilitators had dry run sessions before proper data collection and continued one‐on‐one support. Two facilitators per NGT (one moderator and one note‐taker) were required to run the sessions. Bi‐lingual workers ran NGTs in English (*x* = 4), Arabic (*x* = 5) and Tigrinya (*x* = 1). Three NGTs involved an interpreter or translator. Two of the 10 NGTs were carried out by an independent, experienced senior academic with the participation of trained bilingual workers as part of the capacity building and to ensure compliance with the NGT protocol. The NGTs took 85–100 min to implement due to the complex nature of the process. Each NGT comprised 12 people (range: 6–12). For bowel cancer screening, NGTs were mixed‐sex, whereas for breast cancer and cervical screening, the groups were sex‐specific. Each nominal group lasted about 2.5 h. The NGTs were audio‐recorded and transcribed for analysis. The transcripts were translated into English by accredited translators where needed.

The NGTs' implementation involved four key steps [25]. The silent generation of ideas asked participants to silently list and independently write up to four possible solutions to overcome barriers to and maximise participation in cancer screening programs (Step 1). The facilitator emphasised that there was no right or wrong answer at this stage and asked participants not to consult or discuss their ideas with each other. Generated ideas were collected in rounds (Step 2). Participants may have been tempted to change their answers as responses were read out. So, it was important to reiterate that there was no wrong or right answer, and at this stage, participants were not allowed to change their answers. Each participant was asked to read their first response to the question, and the facilitator would write the idea on the flip chart. However, some participants listed less than four ideas, so the NGT facilitator allowed a participant to pass if they had no new ideas to list in the round. Once ideas were listed, through discussion, participants were asked to identify and delete duplicates (Step 3). Then, participants were asked to identify responses that conveyed the same message or had the same meaning to group or merge them into themes. The discussion of listed ideas gave equal weight to each response and allowed participants to expand an idea, clarify ambiguous responses, or eliminate redundant ideas. Differences of opinion were accommodated, but debate during idea consolidation was managed by focusing on clarification and deferring disagreements to the ranking stage [25]. Finally, participants were asked to choose five most important solutions out of the total from the consolidated list (Step 4). As not all solutions identified as important are amenable to intervention, participants were asked to silently and individually score each identified solution based on their perceived importance (5 = extremely important to 1 = not all important) and perceived easiness to implement (5 = very easy to implement to 1 = very difficult to implement). The importance and easiness of implementing scores for each solution were multiplied to arrive at a total score. Higher scores meant very important and easy to implement; lower scores meant least important and complex to implement.

### 2.4 Data Analysis

Data from 10 participant‐driven NGTs were pooled and analysed [22, 35]. Priorities across NGT sessions were pooled and synthesised through thematic grouping and ranking, enabling identification of shared and divergent priorities. Individual responses were categorised into mutually exclusive priority intervention themes by an independent senior academic and verified by two project management team members, minimising researcher bias and preserving participants' voices. Each original theme retained its score within its assigned priority intervention, with final scores representing the sum of all included items. To account for variation in the number of items across priority interventions, total scores were normalised to a 0–100 range using min‐max normalisation, enabling valid cross‐intervention comparison [36].

## 3 Results

The study included 90 participants from 10 NGTs (Table 1). All NGTs for breast cancer and cervical screening included women only. All group discussions on bowel cancer screening, except one, were gender mixed. Most NGTs had high attendance levels, with group sizes varying from 6 to 12 participants. Participants' mean age and length of stay in Australia varied across groups, from 36.8 to 59.3 years and 4.6 to 19.7 years, respectively. Participants came from culturally and linguistically diverse backgrounds including migrants and refugees from Uganda, Sierra Leone, Burundi, Ethiopia, South Sudan, the Democratic Republic of Congo, Zambia, Zimbabwe, Nigeria, South Africa, Iraq, Syria, Eritrea and Liberia.

**Table 1:** Demographic profile of study participants.

Demographics	African men's group (bowel cancer screening)	African women: (breast cancer screening)	African men and women (bowel cancer screening)	African women (cervical screening)	Arabic women (breast cancer screening)	Arabic men and women (bowel cancer screening)	Arabic women: (breast cancer screening)	African men and women: (bowel cancer screening)	Arabic women: (cervical screening)	African men and women (bowel cancer screening)
Age in years Mean [median; min‐max)	51.4 [51; 41–62]	50.2 [49.5; 41, 58]	46.8 [42; 36–66]	41.5 [39; 28–60]	59.3 [62; 48–68]	59.1 [61; 35–71]	36.8 [39; 27–46]	50.7 [50.5; 40–58]	47 [48; 29, 63]	42 [43.5; 25–57]
Gender (% M/F)	100% M	100% F	66.7% M	100% F	100% F	62.5% M	100% F	30% M 70% F	100% F	50% M 50% F
						37.5% F				
			33.3% F							
Length of stay in Australia in years Mean [median; min–max)	18.0 [19; 6–23]	16.5 [17.5; 5–23]	17.7 [18.5; 8–22]	19.7 [19; 16–24]	11.0 [12; 2–20]	8.3 [8; 1–19]	5.6 [5.5; 1–10]	5.0 [4.5; 4–7]	4.6 [4; 1–9]	6.5 [4.5; 3–12]
Language spoken at home	Krio, Luganda, Dinka, Kirundi, French, Kirundi, Amharic	Nyanga, Bemba	Swahili	Nyanga, bemba, Shona, Ndebele, Setswana, bemba, Alur, Pidgeon, Kalabari	Arabic	Arabic	Arabic	Swahili and Kirundi	Arabic	Tigrinya, Blen, Amharic
			Krio							
			Arabic							
		Shona, Ndebele								
			Temne							
		Setswana								
			Dinka							
		Bemba								
			Mende							
		Alur. Pidgeon								
		Kalabari								
Country of birth	Sierra Leone, Uganda, South Sudan, Ethiopia and Burundi	Zambia, Zimbabwe, South Africa, Uganda, Nigeria	Democratic Republic of Congo, Sierra Leone, South Sudan	Zambia, Zimbabwe, South Africa, Uganda, Nigeria	Iraq and Syria	Iraq and Syria	Iraq, Syria, Tunisia and Turkey	Democratic Republic of Congo and Rwanda	Libya, Syrian and Iraq	Eritrea and Ethiopia
No. of participants in in the group	9	10	12	6	9	8	8	10	9	9

*Cancer literacy knowledge, behaviours and confidence* were identified as the highest‐ranked intervention for bowel cancer screening, founded on three pillars. Enhancing and harnessing community relationships was the highest‐ranked pillar and included eight specific and tailored ranked strategies:

- establishing bowel cancer support groups
- learning about bowel cancer screening from role models and trained community champions
- engaging general practitioners as the primary gateway for routine bowel cancer screening services
- adopting family inclusive approaches that encourage partners' involvement in bowel cancer awareness
- empowering social and refugee‐led community organisations to spread the word about bowel cancer
- maximising the role of social workers as frontline actors in bowel cancer screening delivery and engagement
- initiating community‐based panel discussions and Q&A about bowel cancer, and
- training age‐and gender matched bowel cancer screening educators to address intergenerational issues.

The *promotion of asset‐based community engagement* was the second highest ranked pillar, which emphasised the need to engage with community structures such as schools, technical and further education (TAFE), workplaces and community associations to implement five ranked strategies:

- embedding bowel cancer screening information into pre‐departure cultural orientation and Settlement Engagement and Transition Support Program (SETS)
- incorporating bowel cancer screening education through TAFE
- building bowel cancer screening education into refugee health nurse home visits
- the provision of information about bowel cancer screening to families through school students (e.g., take‐home brochures) and
- mapping and disseminating bowel cancer survivors' testimonies to dispel long‐held myths about bowel cancer.

*Addressing personal level factors related to knowledge, attitudes and skill* as the third highest ranked pillar included three ranked strategies: translating bowel cancer screening messaging into community languages, reducing bowel cancer screening messaging and language complexity, and educating migrant communities about bowel cancer screening through TV programs and social media.

*Policy changes and reform* were identified as being the second highest‐ranked intervention to increase refugees' and migrants' participation in bowel cancer screening programs. Under this intervention package, four strategies were identified and ranked, which were: reducing the age for the National Bowel Cancer Screening Program to 40 years, making bowel cancer screening compulsory, reducing the bowel cancer screening interval from 50 years to every year rather than biennially, and rewarding individuals for undertaking bowel cancer screening.

*Strengthening community action* was identified as being the third highest‐ranked intervention to increase refugees' and migrants' participation in bowel cancer screening programs. Under this intervention package, two strategies were identified and ranked: increasing capacity building and funding of community structures and strengthening community structures and governance.

### 3.1 Bowel Cancer Screening Interventions

Five NGTs were carried out to identify bowel cancer screening strategies that are amenable to intervention (Table 2). Participants identified a total of 24 possible areas of action. Through thematic analysis, these were grouped into four distinct but complementary interventions to increase bowel cancer screening: increasing bowel cancer screening literacy knowledge, behaviours and confidence, policy changes and reform, strengthening community action and monitoring, evaluation, accountability and learning.

**Table 2:** Suggested interventions to increase bowel cancer screening and their ranking.

Proposed interventions for bowel cancer screening: pooled themes and their weightings	No. of NGTs	Raw Total score	Normalised score	Rank
**Increasing bowel cancer literacy knowledge, behaviours and confidence**	5	**833**	761	1
*Enhancing and harnessing relationships: peers and family*	5	397	356	
Cancer support groups: Friends encourage cancer screening	3	104	100	
Learning about bowel cancer from role models and trained community champions	2	68	64	
GP as a focal for bowel for routine cancer screening	4	53	48	
Family inclusive approach: Partner/wife's involvement bowel cancer awareness	1	50	45	
Social and refugee‐led community organisations spreading cancer awareness	3	49	44	
Social workers as frontline actors in bowel cancer screening delivery and engagement	1	36	31	
Community‐based panel discussions and Q&A about bowel cancer	2	18	13	
Age‐and gender matched bowel cancer educators to address intergenerational issues	2	14	9	
Cultural programmes—festivals, celebrations	1	5	0	
*Asset‐based Community Engagement: school, workplace and community structures*	5	229	287	
Embed bowel cancer information into pre‐departure cultural orientation and SETS Program	2	62	100	
Bowel cancer education through TAFE	4	52	74	
Bowel cancer education through refugee health nurse home visits	1	48	63	
Information about bowel cancer for families through school students (take home brochures)	1	43	50	
Bowel cancer survivors' testimonies to dispel myths about bowel cancers	1	24	0	
*Addressing personal level factors related to knowledge, attitudes and skills*	5	207	118	
Translate bowel cancer messaging into community languages	3	129	100	
Reduce bowel cancer messaging and language complexity	2	48	18	
Information and education about bowel cancer through TV programs and social media	3	30	0	
**Policy changes and reform**	**4**	**147**	**217**	**2**
Reduce the age for the national bowel cancer screening program to 40 years	2	61	100	
Making bowel cancer screening compulsory	2	52	83	
Reduce bowel cancer screening after 50 years to every year	1	26	34	
Reward for bowel cancer screening	2	8	0	
**Strengthen community action**	**1**	**118**	**100**	**3**
Capacity building and funding of community structures for bowel cancer education and screening	1	90	100	
Engaging and strengthening community structure and governance for cancer education and screening	1	28	0	
**Monitoring, Evaluation, Accountability and Learning**	**1**	**10**	**10**	**4**
Biannual knowledge, attitude, awareness and practice surveys	1	10	10	

Abbreviation: SETS, settlement engagement and transition support.

*Increasing bowel cancer literacy knowledge, behaviours and confidence* was identified as the highest‐ranked intervention for bowel cancer screening, founded on three pillars. Enhancing and harnessing community relationships was the highest‐ranked pillar and included eight specific and tailored ranked strategies:

- establishing bowel cancer support groups,
- learning about bowel cancer screening from role models and trained community champions,
- engaging general practitioners as the primary gateway for routine bowel cancer screening services,
- adopting family inclusive approaches that encourage partners' involvement in bowel cancer awareness
- empowering social and refugee‐led community organisations to spread the word about bowel cancer
- maximising the role of social workers as frontline actors in bowel cancer screening delivery and engagement
- initiating community‐based panel discussions and Q&A about bowel cancer, and
- training age‐and gender matched bowel cancer screening educators to address intergenerational issues.

The *promotion of asset‐based community engagement* was the second highest ranked pillar, which emphasised the need to engage with community structures such as schools, TAFE, workplaces and community associations to implement five ranked strategies:

- embedding bowel cancer screening information into pre‐departure cultural orientation and Settlement Engagement and Transition Support Program (SETS)
- incorporating bowel cancer screening education through TAFE
- building bowel cancer screening education into refugee health nurse home visits
- the provision of information about bowel cancer screening to families through school students (e.g., take‐home brochures), and
- mapping and disseminating bowel cancer survivors' testimonies to dispel long‐held myths about bowel cancer.

*Addressing personal level factors related to knowledge, attitudes and skill* a the third highest ranked pillar included three ranked strategies: translating bowel cancer screening messaging into community languages, reducing bowel cancer screening messaging and language complexity, and educating migrant communities about bowel cancer screening through TV programs and social media.

*Policy changes and reform* were identified as being the second highest‐ranked intervention to increase refugees' and migrants' participation in bowel cancer screening programs. Under this intervention package, four strategies were identified and ranked, which were: reducing the age for the National Bowel Cancer Screening Program to 40 years, making bowel cancer screening compulsory, reducing the bowel cancer screening interval from 50 years to every year rather than biennially, and rewarding individuals for undertaking bowel cancer screening.

*Strengthening community action* was identified as being the third highest‐ranked intervention to increase refugees' and migrants' participation in bowel cancer screening programs. Under this intervention package, two strategies were identified and ranked: increasing capacity building and funding of community structures and strengthening community structures and governance.

*Monitoring, evaluation, accountability and learning* were identified as being the fourth highest‐ranked intervention to increase refugees' and migrants' participation in bowel cancer screening programs. One strategy was identified under this intervention package: establishing biennial knowledge, attitude, awareness and practice surveys to evaluate the effectiveness of proposed bowel cancer interventions. Implementation would require working with community structures to develop survey tools and training bilingual workers in data collection and dissemination of findings.

### 3.2 Breast Cancer Screening Interventions

Three NGTs were carried out to identify breast cancer screening strategies (Table 3). Participants identified a total of 11 possible areas of action. Through thematic analysis, these were grouped into two distinct but complementary interventions: increasing breast cancer literacy knowledge, behaviours and confidence and policy influences on behaviours. These interventions were ranked according to the number of votes, and strategies were identified and ranked within each intervention.

**Table 3:** Ranked breast cancer screening interventions.

Proposed breast cancer screening interventions: pooled themes and their weightings	No. of NGTs	Raw total scores	Normalised scores	Rank
**Increasing breast cancer literacy knowledge, behaviours and confidence**	3	**333**	352	1
*Enhancing and harnessing relationships: peers and family*	3	155	252	
Themed community events and cultural celebrations	1	58	100	
GP as a focal for breast for routine cancer screening	3	57	98	
Cancer support groups: Friends encourage cancer screening	1	34	54	
Learning about breast cancer from role models and trained community champions	1	6	0	
*Addressing personal level factors related to knowledge, attitudes and skills*	3	223	109	
Community education and awareness raising through workshops and translated materials	3	126	100	
Nutrition education: lifestyle changes and healthy eating	1	52	9	
Dissemination of plain language evidence on early detection and treatment outcome	1	45	0	
**Policy influences on behaviours**	**3**	**296**	299	**2**
Sponsored regular breast cancer check ups	1	152	100	
Reward for breast cancer screening	1	92	53	
Providing transport for those who are unable to travel to test facilities	1	29	5	
Making breast cancer screening compulsory	1	23	0	

Priority interventions for breast cancer screening mirrored those identified for bowel cancer screening. Thus, *increasing breast cancer literacy knowledge, behaviours and confidence* was identified as being the highest‐ranked intervention to increase breast cancer screening uptake. But unlike bowel cancer screening, this intervention had only two pillars. *Enhancing and harnessing relationships within the communities* was the highest‐ranked pillar and contained four ranked strategies:

- themed community events and cultural celebrations
- engaging general practitioners as the primary gateway for routine breast cancer screening services
- establishing breast cancer support groups, and
- learning about breast cancer screening from role models and trained community champions.

*Addressing personal level factors related to knowledge, attitudes and skills* was the second highest‐ranked pillar. It identified three ranked interventions: community education and awareness raising about breast cancer screening through workshops and translated materials, nutrition and health education targeting lifestyle changes and healthy eating and disseminating plain language evidence on early detection and treatment outcome.

*Policy influences on behaviours* were identified as being the second highest‐ranked intervention to increase refugees' and migrants' participation in breast cancer screening programs. Under this intervention package, four ranked strategies were identified:

- sponsored regular breast cancer check‐ups
- reward for undertaking breast cancer screening
- provide transport for those who are unable to travel to and from screening facilities, and
- making breast cancer screening compulsory.

## 4 Cervical Screening Interventions

A total of two NGTs were carried out to identify cervical screening strategies that are amenable to intervention (Table 4). Participants identified a total of eight possible areas of action. Through thematic analysis these were grouped into one distinct intervention—*increasing cervical cancer literacy knowledge, behaviours and confidence*.

**Table 4:** Ranked cervical cancer screening interventions.

Proposed interventions for cervical screening: pooled themes and their weightings	No. of NGTs	Total raw scores	Normalised scores	Rank
**Increasing cervical cancer literacy knowledge, behaviours and confidence**	2	**372**	386	1
*Enhancing and harnessing relationships: peers and family*	2	227	262	
GP as a focal for routine cervical cancer screening	2	64	100	
Themed community events and cultural festivals	2	59	87	
Community‐based panel discussions and Q&A about cervical cancer	1	48	59	
Learning about cervical cancer from role models and trained community champions	1	31	15	
Family inclusive approach: Partner/wife's involvement in cervical cancer screening	2	25	0	
*Asset‐based community engagement: school, workplace and community structures*	2	124	103	
Cervical cancer education through TAFE	2	101	100	
Information about cervical cancer for families through school students (take home brochures)	1	23	3	
*Addressing personal level factors related to knowledge, attitudes and skills*	2	21	21	
Information and education about cervical cancer through TV programs and social media	2	21	21	

This intervention was founded on three ranked pillars*. Enhancing and harnessing relationships within the communities* was the highest‐ranked pillar and included five ranked strategies:

- engaging General Practitioners (GPs) as the primary gateway for routine cervical screening services
- themed community events and cultural festivals
- community‐based panel discussions and Q&A about cervical screening
- learning about cervical screening from role models and trained community champions, and
- promoting a family‐inclusive approach that emphasises partners' involvement in cervical screening.

*Asset‐based community engagement* was the second highest ranked pillar and emphasised the need to engage community assets such as schools, TAFE, most popular workplaces for refugees (e.g., factories), and community associations. Under this pillar, two strategies were identified:

- cervical screening education through TAFE, and
- information about cervical screening for families through school students (take‐ home brochures).

*Personal level factors related to knowledge, attitudes and skills* were the third highest‐ranked pillar, which contained one strategy‐ disseminating information and education about cervical cancer through TV programs and social media.

## 5 Discussion

This novel intervention‐ranking study sought to identify and prioritise effective cancer screening strategies to enhance migrant and refugee communities' participation in the national cancer screening programs. It identified community‐informed interventions, thereby shaping the agenda on what works, for whom and under what circumstances. This study is timely as available data point to cancer prevention programs prioritising the host country's mainstream language in most high‐income countries, including Australia, over migrant community needs [37]. The under‐representation of migrant and refugee communities in cancer screening programs represents a serious challenge in Australia. It exacerbates inequities in access, resulting in reduced cancer screening participation and poor cancer survival [29, 30, 38]. Failing to close the migration‐related cancer‐equity gap in screening and beyond may lead to misallocation of funds (i.e., failing to invest where the needs are highest). In the broader context, approximately $200 billion is spent globally each year on health and medical research and interventions [39, 40, 41]. Of this, around 85% (about $170 billion) is considered wasted due to poorly designed interventions or research focusing on interventions addressing questions and outcomes of limited relevance to some communities [39, 40, 41]. Co‐designing cancer screening interventions has the potential to increase migrant and refugee communities' involvement in implementing these screening programs and increasing their uptake.

Increasing cancer literacy emerged as a common thread across all three screening programs. Evidence indicates that cancer literacy among migrant and refugee communities, spanning primary, secondary and tertiary prevention, is substantially lower than in host populations [37], and is often compounded by traditional beliefs and myths [42]. Myths identified during idea consolidation in our study included beliefs that cancer only affects older people, that screening is unnecessary without symptoms, that cancer is linked exclusively to family history, that screening is painful or invasive, and that cancer is always fatal rendering screening futile. Bowel cancer‐specific myths included negative perceptions around handling one's own faeces, and beliefs that screening signals weakness, undermines masculinity or implies impotence. These findings align with the broader literature, which additionally documents beliefs that cancer is contagious, a divine punishment or invariably a death sentence [42, 43]. Collectively, such myths pose a significant barrier to timely, potentially life‐saving screening participation.

Our findings suggest that increasing cancer literacy may need to incorporate activities that build on and empower community relationships, structures and assets and address personal level factors related to knowledge, attitudes and skills. Several pilot studies examining how to reduce cancer disparities among migrants through English language programs or education curricula have produced robust results in increasing cancer literacy [44, 45]. Similar promising results have been reported when increasing cancer literacy through community settings such as places of worship (faith‐based cancer education and lifestyle interventions) [46, 47, 48, 49], using layperson community health champions and other community members either as volunteers, role models or paid community health workers [50], and themed community events and cultural celebrations [51, 52, 53].

Addressing personal‐level factors related to knowledge, attitudes and skills remains a persistent challenge [42, 43]. Cancer‐related myths, beliefs and stigma are rarely addressed within existing screening programs, undermining efforts to raise awareness and shift individual behaviours. Low cancer literacy reduces the likelihood that individuals will adopt cancer‐risk‐reducing behaviours or seek support following diagnosis [42]. While effective, accessible and culturally responsive health systems in host countries can reduce the impact of such myths, exploratory research in this area remains limited in Australia. A Korean trial [54] examining community outreach and clinic‐ and pharmacy‐based strategies for breast cancer reported a 20 and 19 percentage‐point reduction in myths about the link between cancer and breast size and mammography costs respectively, which translated into a 14 percentage‐point increase in intention to undergo screening mammography, demonstrating that targeted myth‐busting interventions can meaningfully shift screening behaviours.

Another common thread across the three cancer screening programs was policy influences. Implicit in this intervention component are the incremental shifts in existing cancer screening (policy change, e.g., making the screening compulsory or rewarding those who get screened) and significant policy changes (reform, e.g., revisiting the eligibility criteria). It is worth acknowledging that policy changes and reform are not easy to achieve because of the different levels of governance guiding cancer service delivery, the maturity of the evidence to inform policy change, different stakeholders' perspectives and the political context and legislative landscape [55, 56].

While the National Preventive Health Strategy 2021–2030 seeks to align action through whole‐of‐government reform, meeting the needs of migrants and refugees remains difficult due to intersecting challenges: population heterogeneity that resists blanket approaches; structural barriers including cost, eligibility and transport; language and communication gaps; low trust shaped by prior health system experiences; culturally embedded beliefs and stigma; service fragmentation; limited culturally competent workforce capacity; and competing settlement priorities that displace preventive health. Together, these factors weaken the translation of policy intent into equitable cancer prevention outcomes, and may hamper implementation of the priorities identified in this study, particularly where policy change requires lengthy and complex engagement processes to establish community benefit and buy‐in.

Migrant and refugee communities emphasised strengthened community actions to increase participation in cancer screening programs. Okasako‐Schmucker et al. [57] have detailed strategies for improving community actions that have effectively increased breast, cervical and bowel cancer screening. The first strategy is increasing the demand for screening services by adopting culturally appropriate group education. The primary goal of group education is to overcome barriers to screening by emphasising indications for and benefits of seeking recommended screening. To be effective, group educators must be from the target communities; and trained lay people who then contribute towards building the capacity within their respective communities. The second‐dimension centres on customised one‐on‐one education on the benefits of cancer screening recommendations, preferably delivered by lay health advisors or volunteers recruited from the target communities. In turn, these human community assets can be used to coordinate cancer screening reminders and/or assist community members with scheduling appointments. They can also be used in community‐based small media strategies, including distributing videos and printed materials, such as letters, brochures and newsletters about cancer in community languages tailored to specific individuals or communities. Finally, strengthening community actions may require addressing structural barriers that make it difficult for migrants to access cancer screening programs. Strategies may include involving community volunteers to reduce time or distance between screening sites and target populations, modifying hours of service delivery to meet migrants' needs, offering screening at non‐clinical community settings, such as mobile mammography vans at worksites or in residential communities, and eliminating administrative procedures and other obstacles such as the use of suitably trained community bilingual workers to complement professional interpreters, when appropriate to do so.

## 6 Strengths and Limitations

Data were drawn from a wide range of migrant groups across diverse ethnicities, with thematic grouping demonstrating consistency in cancer screening priorities across groups, strengthening the robustness of findings. However, as recruitment was conducted through partner organisations delivering settlement services, participants are more likely to represent those already engaging with such services, which may limit generalisability to the broader migrant population. This nonetheless provides valuable, context‐specific insight into how settlement support interfaces with health system engagement and offers a baseline for understanding how cancer prevention messages are received within the settlement context. Further research is needed that includes more diverse migrant cohorts beyond settlement service users, including skilled and family migrants and temporary residents transitioning to permanent residency.

## 7 Conclusion

Improving migrant and refugee participation in bowel, breast and cervical cancer screening requires a dual focus: strengthening individual agency and enacting supportive structural and policy reforms. Across all three cancer types, findings consistently point to the need for culturally grounded, community‐led approaches operating at both the personal and environmental level. At the individual level, priority must be given to building cancer literacy, knowledge, attitudes and confidence. This includes embedding cancer awareness into settlement pathways such as the Adult Migrant English Program, SETS and pre‐departure cultural orientation. Plain‐language translated materials and simplified messaging are essential across all three screening programs. Cancer survivor testimonies, trained community champions and peer‐to‐peer learning offer credible, culturally appropriate motivation for uptake. GP relationships as a routine screening referral point, alongside family‐inclusive approaches involving partners and spouses, further reinforce individual decision‐making. At the environmental level, asset‐based community engagement through schools, workplaces, TAFE, cultural festivals and refugee‐led organisations provides a ready infrastructure for sustained cancer education. Social workers and refugee health nurses serve as frontline outreach actors, while community panel discussions, Q&A forums and age‐ and gender‐matched educators address intergenerational and gender‐specific barriers. Policy reform is integral to improving screening equity. Key recommendations include reducing the eligible age for bowel cancer screening to 40 years, increasing bowel screening frequency to annually after age 50, introducing incentive mechanisms for bowel and breast cancer screening, and providing sponsored transport to address access barriers. Consideration should be given to making participation in bowel and breast cancer screening compulsory. Sustained progress requires dedicated capacity building and funding of community structures, strengthened community governance, and biannual knowledge, attitude, awareness and practice surveys to monitor outcomes and ensure interventions remain responsive across all three cancer programs.

## Author Contributions

**Andre M. N. Renzaho:** conceptualisation, investigation, methodology, validation, visualisation, software, formal analysis, data curation, supervision, resources, writing – original draft, writing – review and editing. **Sheetal Challam:** conceptualisation, investigation, funding acquisition, writing – review and editing, methodology, validation, visualisation, supervision, formal analysis, resources. **Nina Hartcher:** conceptualisation, investigation, funding acquisition, writing – review and editing, validation, visualisation, supervision, resources. **Mitchell Smith:** writing – review and editing, resources, conceptualisation, validation. **Sandra Elhelw:** writing – review and editing, resources, investigation, validation. **Amanda Jayakody:** investigation, writing – review and editing, resources, funding acquisition. **Sarah McGill:** investigation, funding acquisition, writing – review and editing, resources, validation. **Tracey A. O'Brien:** conceptualisation, investigation, funding acquisition, writing – review and editing, validation, visualisation, supervision, resources, data curation, methodology.

## Ethics Statement

South‐Western Sydney Local Health District Human Research Ethics Committee (2022/ETH01516).

## Consent

All authors have read and agreed to the published version of the manuscript.

## Conflicts of Interest

The authors declare no conflicts of interest.

## Data Availability

The data that support the findings of this study are available on request from the corresponding author. The data are not publicly available due to privacy or ethical restrictions. All data are available on paper.
